# Self-Administered Behavioral Skills–Based At-Home Virtual Reality Therapy for Chronic Low Back Pain: Protocol for a Randomized Controlled Trial

**DOI:** 10.2196/25291

**Published:** 2021-01-19

**Authors:** Laura M Garcia, Beth D Darnall, Parthasarathy Krishnamurthy, Ian G Mackey, Josh Sackman, Robert G Louis, Todd Maddox, Brandon J Birckhead

**Affiliations:** 1 Research and Development AppliedVR Inc Los Angeles, CA United States; 2 USC Creative Media and Behavioral Health Center Los Angeles, CA United States; 3 Department of Anesthesiology, Perioperative and Pain Medicine Stanford University School of Medicine Stanford, CA United States; 4 C T Bauer College of Business Houston, TX United States; 5 Division of Neurosurgery Pickup Family Neurosciences Institute Hoag Memorial Hospital Newport Beach, CA United States; 6 Division of Health Services Research Department of Medicine Cedars-Sinai Health System Los Angeles, CA United States

**Keywords:** chronic pain, virtual reality, behavioral medicine, behavioral health, pain treatment, randomized controlled trial

## Abstract

**Background:**

Chronic pain is one of the most common and debilitating health conditions. Treatments for chronic low back pain typically focus on biomedical treatment approaches. While psychosocial treatments exist, multiple barriers prevent broad access. There is a significant unmet need for integrative, easily accessible, non-opioid solutions for chronic pain. Virtual reality (VR) is an immersive technology allowing innovation in the delivery of behavioral pain treatments. Behavioral skills-based VR is effective at facilitating pain management and reducing pain-related concerns. Continued research on these emerging approaches is needed.

**Objective:**

In this randomized controlled trial, we seek to test the efficacy of a self-administered behavioral skills-based VR program as a nonpharmacological home-based pain management treatment for people with chronic low back pain (cLBP).

**Methods:**

We will randomize 180 individuals with cLBP to 1 of 2 VR programs: (1) EaseVRx (8-week skills-based VR program); or (2) Sham VR (control condition). All participants will receive a VR headset to minimize any biases related to the technology’s novelty. The Sham VR group had 2D neutral content in a 3D theater-like environment. Our primary outcome is average pain intensity and pain-related interference with activity, stress, mood, and sleep. Our secondary outcomes include patient-reported physical function, sleep disturbance, pain self-efficacy, pain catastrophizing, pain acceptance, health utilization, medication use, and user satisfaction. We hypothesize superiority for the skills-based VR program in all of these measures compared to the control condition. Team statisticians blinded to treatment assignment will assess outcomes up to 6 months posttreatment using an approach suitable for the longitudinal nature of the data.

**Results:**

The study was approved by the Western Institutional Review Board on July 2, 2020. The protocol (NCT04415177) was registered on May 27, 2020. Recruitment for this study was completed in July 2020, and data collection will remain active until March 2021. In total, 186 participants were recruited. Multiple manuscripts will be generated from this study. The primary manuscript will be submitted for publication in the winter of 2020.

**Conclusions:**

Effectively delivering behavioral treatments in VR could overcome barriers to care and provide scalable solutions to chronic pain’s societal burden. Our study could help shape future research and development of these innovative approaches.

**Trial Registration:**

ClinicalTrials.gov NCT04415177; https://clinicaltrials.gov/ct2/show/NCT04415177

**International Registered Report Identifier (IRRID):**

RR1-10.2196/25291

## Introduction

Chronic pain is one of the most common reasons adults seek medical care [1]. Chronic pain affects between 50 and 116 million Americans, more than cancer, diabetes, and cardiovascular disease combined [1-4]. Other estimates suggest that 25 million American adults live with moderate to severe chronic pain (ie, pain scoring 4-7 on a visual analog scale and lasting over 3 months) that limits their activities and diminishes their quality of life [5,6]. Because of this great need, it is imperative to develop and test effective treatments for chronic pain.

Pain treatment and management often emphasize biomedical approaches, such as pharmacology or surgical procedures. Historically, opioids were commonly prescribed for pain treatment and management. These agents can yield both inconsistent and suboptimal results [7] and carry numerous personal and public health risks. The Centers for Disease Control and Prevention (CDC), the Centers for Medicare & Medicaid Services (CMS), and the Department of Health and Human Services recommended nonpharmacological modalities as first-line treatments for pain, including behavioral treatments [8,9]. Low-risk behavioral treatments may facilitate improved outcomes and analgesia while minimizing health risks.

Indeed, evidence-based behavioral treatments are effective for treating chronic pain. Therapies such as cognitive behavioral therapy for chronic pain, mindfulness-based stress reduction [10,11], and acceptance and commitment therapy [12] have been shown to modify cognitions and behaviors that influence the perception of pain. Although behavioral therapies show some promise, multiple barriers prevent chronic pain patients from accessing these behavioral treatment alternatives [13]. Strict reliance on skilled therapists that are in short supply, travel burdens, long durations of treatments, inadequate insurance coverage, and high costs can all contribute to a lack of treatment accessibility and patient engagement [14-16]. Furthermore, almost 85% of patients do not report meaningful analgesia from their pain medications (ie, they do not experience a long-term ≥50% reduction in their pain levels) [17]. Therefore, there is an urgent need for effective and comprehensive solutions for chronic pain and behavioral treatment delivery methods that are accessible to the entire spectrum of individuals affected by this concern.

Digital therapeutics for chronic pain are cost-effective, available on-demand, can be delivered in the home, and improve the risk–benefit profile well above the current standard of care. In particular, virtual reality (VR) therapeutics show promise as effective treatments for acute and chronic pain [18-24]. With the first pain reduction VR program, SnowWorld, patients with pediatric burn undergoing physical therapy noted a 27%-44% reduction in pain (*P*<.05) in comparison to within-subject control [25]. To date, VR has been used in numerous clinical settings to reduce pain and improve outcomes in complex regional pain syndrome [26], chronic headache/migraine pain [27], fibromyalgia [28,29], and chronic musculoskeletal pain [30]. Technology allows for an immersive, multisensory, and interactive virtual treatment experience. By stimulating the visual, auditory, and proprioception senses, VR facilitates distraction to limit the user’s processing of nociceptive stimuli, which has been shown in functional magnetic resonance imaging studies [31]. Most importantly, VR therapeutics have the potential to enhance pain education and effectively deliver evidence-based behavioral interventions.

A randomized clinical trial recently examined the effectiveness of a 21-day skills-based VR program for chronic pain compared to the same content delivered in audio form [32]. The VR skills-based program was superior in improving pain intensity and pain-related interference with activity, sleep, mood, and stress compared to the audio-based treatment, with results strengthening after 2 weeks. Results suggested that VR’s immersive components enhanced VR participants’ outcomes relative to those who completed an audio treatment [31]. Nevertheless, it is unclear to what extent these positive outcomes were due to the VR technology’s novelty and whether VR effects are durable. Therefore, this study seeks to conduct a randomized controlled trial to test the effectiveness of a comprehensive 56-day behavioral skills–based VR therapeutic program (skill-based VR) in chronic low back pain (cLBP). This study will elucidate the immediate and long-term effects of this proposed treatment while comparing it to a nontherapeutic control condition designed to account for this technology’s novelty.

We hypothesize that therapeutic VR will significantly benefit self-reported pain intensity and pain-related outcomes compared to our control condition throughout this 8-week treatment and follow-up period. This study will address the following 4 objectives:

- The primary objective is to assess the impact of skills-based VR on changes in patient-reported pain and pain interference throughout an 8-week intervention and in comparison to a placebo VR condition.
- The secondary objective is to assess the impact of skills-based VR on changes in patient-reported satisfaction (Patient’s Global Impression of Change [PGIC]) throughout an 8-week intervention and in comparison to a placebo VR condition.
- The tertiary objective is to assess the impact of skills-based VR on changes in patient-reported opioid use, physical function, pain coping, and health outcomes immediately following the intervention relative to a preintervention baseline and in comparison to a placebo VR condition.
- The exploratory objective is to assess the impact of skills-based VR on changes in patient-reported pain levels, opioid use, physical function, pain coping, health outcomes, and satisfaction for 6 months following intervention and in comparison to a placebo VR condition.

## Methods

### Overview

**Figure 1:**
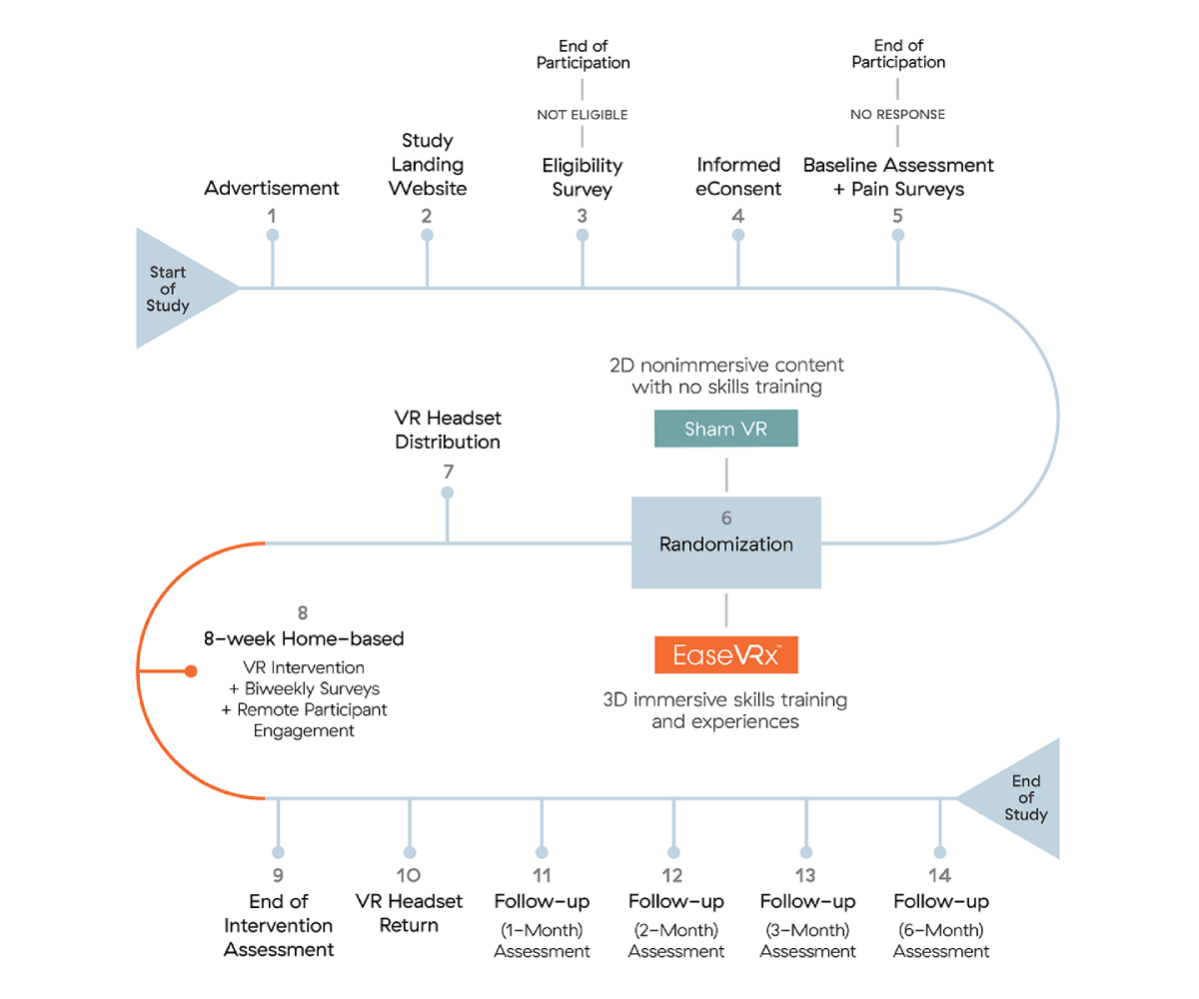
Timeline of protocol activities: This figure depicts each of the steps that participants in this study will go through, starting from the moment they receive an advertisement for the study until the end of our follow-up assessments.

We will conduct a single-cohort, double-blinded (participant and analysts), cross-sectional, placebo-controlled randomized clinical trial in which 180 community-based individuals with cLBP will be randomly assigned to a 56-day skills-based VR therapeutic program (EaseVRx) and a 56-day control VR condition (Sham VR). Participants will be followed for 8.5 months after randomization. Participant eligibility will be assessed with an electronic screener survey. Once enrolled in the study, participants will complete a 2-week baseline assessment period, an 8-week VR program, a posttreatment assessment, and up to 4 posttreatment follow-ups over 6 months. During their 2-week baseline period, participants will be required to complete their baseline assessment and at least one of three pain surveys in order to progress to the treatment phase of the study in which they will receive a VR headset with their assigned treatment to be completed at home (Figure 1).

Team statisticians blinded to participant treatment assignment will examine outcomes immediately following treatment and after 1, 2, 3, and 6 months following treatment. The 6-month postintervention assessment is exploratory. Our primary outcome will be average pain intensity and pain interference on activity, sleep, mood, and stress. Our secondary outcomes include self-reported change in average pain intensity, physical function, sleep disturbance, pain catastrophizing, pain self-efficacy, pain acceptance, skills use, health utilization, medication use, and treatment usage and satisfaction.

The protocol for this trial has been approved by the Western Institutional Review Board. All participants will be required to give their informed consent during their online screening before enrollment in the study.

### Study Sample, Setting, and Recruitment

Community-based individuals with cLBP will be recruited nationally through chronic pain organizations (eg, American Chronic Pain Association) and advertising on social media platforms such as Facebook and Twitter. Additionally, study advertisements will be emailed to professional contacts at several medical clinics with requests to forward among medical colleagues nationally. All advertisements will direct interested individuals to a landing page where detailed study information exists. Interested individuals will be directed to complete an online REDCap Cloud (nPhase, Inc.) screening form to assess their eligibility.

The online screening will automatically classify individuals as eligible or ineligible using survey logic based on our inclusion/exclusion criteria. Ineligible individuals will receive a message thanking them for their interest and participation in the screening process, inform them of their ineligibility, and be given a list of chronic pain resources. Eligible individuals will be redirected to an electronic consent form to provide their signature and complete enrollment.

Enrolled participants will progress to the study’s treatment phase if they complete a baseline survey and at least one of three pain surveys during the 2-week baseline period. Following the 2-week baseline period, participants will be randomized to a treatment group. The study will enroll 180 adults (age 18-85 years) with cLBP who meet study criteria (Textbox 1). This sample size accounts for expected attrition.

### Inclusion and Exclusion Criteria

Textbox 1 lists the inclusion and exclusion criteria. The reason that radicular symptoms were excluded was to create a degree of homogeneity within the population recruited. Chronic lower back pain with radicular symptoms is often treated differently from those that do have those symptoms. Additionally, we require that participants be willing and available to participate during the study (8.5 months). Participants were asked to complete biweekly surveys during the 56-day treatment to which they are assigned and complete the posttreatment follow-up assessments (1, 2, 3, and 6 months).

**Inclusion and exclusion criteria. DVPRS: Defense and Veterans Pain Rating Scale; VR: virtual reality.**

**Inclusion criteria**

1. Men and women aged 18-85.
2. Diagnosis of low back pain without radicular symptoms.
3. Pain duration of at least six months.
4. Average pain intensity of ≥4 on the 0-10 DVPRS Pain Scale for the past month at screening.
5. English fluency.
6. Willing to comply with study procedures/restrictions.
7. Access to Wi-Fi.

**Exclusion criteria**

1. Unable to understand the goals of the study due to cognitive difficulty.
2. Current or prior diagnosis of epilepsy, seizure disorder, dementia, migraines, or other neurological diseases that may prevent the use of VR.
3. Medical condition predisposing to nausea or dizziness.
4. Hypersensitivity to flashing light or motion.
5. No stereoscopic vision or severe hearing impairment.
6. Injury to eyes, face, or neck that prevents comfortable use of VR.
7. Cancer-related pain.
8. Moderate level of depressive symptoms (subclinical) as indicated by the Patient Health Questionnaire-2 (PHQ) [33,34] depression screen score of >2.
9. Previous use of EaseVRx for pain.
10. Current participation in any interventional research study or completed participation in the past 2 months.
11. Currently pregnant or planning to become pregnant during the study period.
12. Does not have access to Wi-Fi during participation in the study.
13. Currently works at or has an immediate family member who works for a digital health company or pharmaceutical company that provides treatments for acute or chronic pain.

### Randomization and Blinding

Enrolled participants will be randomized 1:1 and assigned to 1 of 2 treatment arms: a 56-day skills-based VR program (EaseVRx) and a 56-day control VR condition (Sham VR). Random assignment will rely on REDCap Cloud’s automatic program to ensure blinded randomization and equal numbers in both treatment arms. This will be a double-blinded study wherein participants and statisticians will be blinded to treatment. An independent research coordinator will label each group as Group A and Group B randomly before sending any data sets to the statistician. Three staff members (LG, IM, and BB) will be unblinded to the treatment groups and will not be involved in any data analyses.

### Study Interventions

Participants in both the EaseVRx and Sham VR conditions will receive a Pico G2 4K headset with either EaseVRx or Sham VR condition. These devices will be mailed to the participant’s self-reported address. Study staff will monitor participant progress through twice-weekly surveys of device use and provide guided technical support. The following sections describe the components of the study interventions.

### VR Headset and Software

This study will use a Pico G2 4K all in one head–mounted display that delivers VR images and sounds. We selected the Pico G2 4K because it is commercially available, widely used, inexpensive, has minimal visual latency, and is much easier for participants to use than many other devices. The user’s exhale, a major mechanic of the EaseVRx program, is measured by the microphone embedded in the Pico G2 hardware, offering biodata-enabled immersive therapeutics. This hardware allows for displaying 3D images (EaseVRx) and 2D images (Sham VR).

#### Skills-Based VR (EaseVRx)

Participants randomized and allocated to this treatment arm will receive a multimodal, skills-based, self-management VR program, called EaseVRx (AppliedVR), that incorporates evidence-based principles of cognitive behavioral therapy and mindfulness. Developed by AppliedVR in partnership with a pain psychology expert, the program provides pain neuroscience education and trains users on evidence-based pain and stress management strategies via immersive and enhanced biofeedback experiences. EaseVRx combines biopsychosocial pain education, diaphragmatic breathing training, relaxation exercises, and executive functioning games to provide a mind–body approach toward living better with chronic pain. The standardized 56-day program delivers a multifaceted combination of skills training through a prescribed sequence of daily virtual experiences. Each VR experience lasts between 2 and 16 minutes, with an average duration of 6 minutes of treatment time. The VR treatment modules were designed to minimize triggers of emotional distress or cybersickness. These modules include:

- Interoceptive modules: biofeedback-like environments that shift in nature to reflect a progressively enhanced state of relaxation.
- Education modules: visually guided lessons explain why the VR exercises are relevant to their pain and specific topics relevant to behavioral medicine for pain.
- 360 video modules: high-quality 360 videos with voiceovers, music, breathing effects, and sound effects that are designed to maximize relaxation and participant engagement.
- Game modules: games are designed to maximize immersive distraction to decrease their perception of pain.
- Dynamic breathing modules: interactive virtual worlds where the user experiences a gamified biofeedback session and is introduced to awareness of their breath via visualization. These modules become increasingly challenging to better train participants in the practice of diaphragmatic breathing.

#### Sham VR

**Figure 2:**
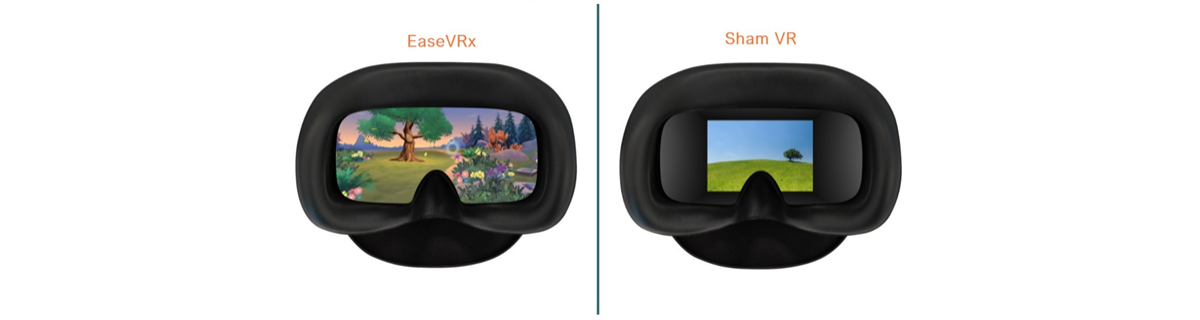
Interventions: This figure depicts the Skills-Based VR condition and control VR condition.

VR-CORE guidelines suggest using an active control in VR clinical trials and promoting nonimmersive, 2D content within a VR headset as an optimal placebo [23]. Thus, participants in the Sham VR group will receive the same Pico G2 4K headset as participants in the immersive VR groups, but instead of 360-degree, 3D, interactive content specially selected for efficacy, they will only view 2D nature footage with neutral music layered on top that is selected to be neither overly relaxing nor distracting. The experience of Sham VR is similar to watching a large-screen TV. The content that is displayed in the VR sham will be viewed in a void theater. The void theater will consist of a solid black environment with the 2D content displayed on a “screen” in front of the user. The screen will take up a significant portion of the field of view of the participant, but appear to be distant enough to minimize any sense of immersion caused by viewing 3D content. The void theater screen will be fixed in place such that the user is capable of looking away from the screen if they so choose. The content for the VR sham will be 2D stock nature videos, all displayed in the void theater. The videos have been chosen to be more distracting than relaxing, and the majority of them contain animals engaging in play, grazing, grooming, or other inoffensive behaviors. There will be 20 videos that will be rotated over the 56 sessions, with a duration between 2.5 and 5 minutes, which corresponds directly with durations in the EaseVRx program. Figure 2 provides a visualization of the kinds of content each VR program would provide.

### Technical Support

Participants will be provided with onboarding material as well as emails describing the study procedures and details. Instructional videos will be made available to participants, and access to remote technical support will be provided. VR usage data for both treatment groups will be surveyed twice a week for the intervention’s 8-week duration.

Survey data will be monitored for completion and technical support staff will be available. Participants will receive a telephone number and email address to contact support staff as needed. The technical support staff will also reach out if there is low adherence to the devices, lack of survey data, or low battery power detected on the headset’s remote monitoring dashboard. Twice weekly, the research staff will review the REDCap Cloud survey dashboard to assess if participants are completing the study. If a survey is missed, the REDCap Cloud system will send up to 2 reminders 24 hours apart. If the participant does not respond to the reminders, a research staff member will send an email or SMS text message to understand why there has been no response and encourage them to re-engage with the study. If the survey remains incomplete after 2 weeks of no data, the participant will be deemed lost to follow up. The participant could come back to the study at any time.

### Study Measures

This section details the measurement and methods used to assess each variable. Table 1 outlines the categories, name, rank, and number of items for all measures. The time interval for collecting these measures is provided in Table 2.

#### Demographics

Demographic variables will include age, gender, level of education, race, ethnicity, employment status, annual household income, relationship status, duration of back pain (years since onset), state of residence, and zip code. In order to perform geospatial coding, rural–urban commuting area codes will be downloaded from a public data set provided by the United States Department of Agriculture Economic Research Service. Using MS Excel, participant zip codes will be matched to the rural–urban commuting area data set to classify participants living in rural or urban areas.

#### Average Pain Intensity

The Defense and Veterans Pain Rating Scale (DVPRS) [35] will be used to measure average pain intensity over the previous 24 hours using an 11-point numeric rating scale (0=no pain; 10=as bad as it could be; nothing else matters).

#### Pain Interference With Activity, Mood, Sleep, and Stress

The DVPRS interference scale (DVPRS-II) will be used to measure pain interference with activity, sleep, mood, and stress over the past 24 hours [36] (0=does not interfere; 10=completely interferes).

#### Patient Global Impression of Change

Aligning with IMMPACT (Initiative on Methods, Measurement, and Pain Assessment in Clinical Trials) recommendations for pain research [37,38], Patient Global Impression of Change will be assessed using the question, “Since the beginning of VR treatment, how would you describe the changes (if any) in activity limitations, symptoms, emotions and overall quality of life-related to your low back pain?” on a 7-point scale ranging from 1 (No change or condition is worse) to 7 (A great deal better, and a considerable improvement that has made all the difference).

#### Physical Function and Sleep Disturbance (PROMIS)

The NIH Physical Function and Sleep Disturbance (PROMIS) [39] short-form measures will be used to assess physical function (version 6b [40]) and sleep disturbance (version 6a [41]) over the past 7 days. Higher scores on physical function signify greater function, whereas higher scores for sleep disturbance reflect greater symptom severity. The conversion table within the scoring manuals, made available from the Person-Centered Assessment Resource [39,42], will be used to calculate the individual short-form T scores using the Item Response Theory scoring algorithms. Specifically, based on published item parameters, T scores (latent trait estimates) will be computed for each individual’s response pattern using the Bayesian expected a posteriori method [43-45]. This has been widely applied within pain research [35-37,39-41,43-48].

#### Pain Catastrophizing

The 13-item Pain Catastrophizing Scale (PCS) [49] is a validated instrument widely used clinically and in pain research to assess patterns of negative cognition and emotion in the context of actual or anticipated pain. Despite having discrete subscales for rumination, magnification, and feelings of helplessness related to pain, prior work has shown that the PCS operates unidimensionally [50] and Cook et al (unpublished). Aligning with prior work [32] and the goal of brevity, the following 4 PCS items will be used: “It’s terrible and I think it’s never going to get any better,” “I become afraid that the pain will get worse,” “I can’t seem to keep it out of my mind,” and “I keep thinking about how badly I want the pain to stop.” Respondents rate the frequency with which they experience such thoughts on a scale from 0 (Not at all) to 4 (All the time). The 4 numerical ratings will be summed to create a total score and index for pain catastrophizing.

#### Pain Self-Efficacy

Pain Self-Efficacy was assessed in 2 ways. First, the 2-item Pain Self-Efficacy Questionnaire (PSEQ-2) will be administered as a validated instrument used to assess respondents’ confidence in their ability to engage in various daily activities despite their chronic pain [51]. The PSEQ-2 consists of the following 2 items: “I can still accomplish most of my goals in life, despite the pain”, and “I can live a normal lifestyle, despite the pain.” Respondents will use a 7-point scale to rate their response from 0 (Not at all confident) to 4 (Completely confident). Scores for the 2 items are summed to create a total score. Second, participants will be asked to rate their overall confidence in their ability to manage their pain on a 10-point scale with 1 (Not at all Confident) to 10 (Very Confident). Following the intervention, this section will be divided into 2 items measuring their overall confidence levels while inside of VR and outside of VR.

#### Chronic Pain Acceptance

The Chronic Pain Acceptance Questionnaire (CPAQ-8) short form is an 8-item validated instrument that assesses one’s engagement in personally meaningful activities despite pain, as well as efforts directed at controlling pain (example item: “I am getting on with the business of living no matter what my level of pain is”) [52]. Respondents rate each item using a 6-point scale ranging from 0 (never true) to 5 (always true).

#### Device Utilization

The custom device utilization survey is a single-item instrument that assesses the number of VR sessions completed since the last time it was asked. Respondents select either (1) 0, (2) 1, (3) 2, (4) 3, or (5) 4 or more. This survey is administered on a biweekly basis.

#### System Usability Scale

The System Usability Scale is a validated, 10-item attitude Likert scale giving a global view of subjective assessments of usability (example item: “I thought the system was easy to use.”) [53]. Participants rate each item using a 5-point response scale ranging from "Strongly Disagree" to "Strongly Agree." Some items are reverse scored, a multiplier is applied to the sum total, and total SUS scores range from 0-100.

#### Immersive Tendencies Questionnaire

The Immersive Tendencies Questionnaire (ITQ) is a 29-item survey that measures difference in tendencies of individuals to experience presence [54]. The involvement subscale was chosen by the coauthors to reduce participant burden with just 7 items that focus on propensity to be engaged with content such as reading a book or watching a movie.

#### Assessment of Affect

The Positive and Negative Affect Schedule (PANAS) is a validated 20-item survey to assess the affect of each participant [55]. They will be asked to the extent they have felt specific emotions on a Likert scale from 1 “Very Slightly or Not at All” to 5 “Extremely.”

#### Prescription Opioid and Analgesic Medication Use

A custom survey was also created to assess analgesic medication use. The medication survey consists of 3 main questions to assess for the use of the following: prescription medication, over-the-counter medication, or other medications. Prescription opioid data will be converted to a standardized morphine milligram equivalent daily dose using the Centers for Medicare & Medicaid Services “Opioid Oral Morphine Milligram Equivalent (MME) Conversion Table” [56]. Endorsement of prescription medications will prompt additional items to collect the type of medication, frequency of use, dose, happiness with one’s current prescribed medication regimen, and interest in changing one’s current prescribed medication regimen.

#### Health Care Utilization

We will also assess cLBP health care utilization in terms of frequency of steroid injections, lower back surgery, emergency department visits, hospital admissions, and unplanned physician visits over various periods.

#### Additional Custom Surveys

Several custom surveys were developed for the study, including one designed to assess satisfaction with each condition. Another assesses device usability, enjoyment or difficulties, and the likelihood to continue treatment. Additional items will assess pain knowledge and pain management skills use (eg, use of relaxation and controlled breathing during the previous 7 days). We will also assess patient perception of the study arm using a single item administered to both groups in the 6-month follow-up survey.

**Table 1:** Variable/category, measure name, rank, and number of items for all measures.

Variable or Category		Measure	Number of items or units
**Primary Outcome**			
	Pain intensity/Pain interference (activity, mood, sleep, stress)	DVPRS-I^a^ Pain Scale and DVPRS-II Pain Scale Measures [36]	5
**Secondary Outcomes**			
	Global impression of change	Patient’s Global Impression of Change (PGIC) [38]	1
	Physical function	PROMIS^b^ Physical Function [40]	6
	Sleep disturbance	PROMIS Sleep Disturbance [41]	6
	Acceptability	Custom Patient Satisfaction	8 + 15 open-ended questions
	Adherence	Custom Device Utilization survey	1
	Adherence	VR^c^ usage data	Seconds/week
	Pain self-efficacy	Pain Self-Efficacy Questionnaire (PSEQ) [51] (general) and Custom Pain Self-Efficacy Questionnaire with VR as a referent (inside the VR headset and outside the VR headset)	2 in each
	Pain acceptance	Chronic Pain Acceptance Questionnaire (CPAQ) [52]	8
	Pain catastrophizing	Pain Catastrophizing Scale (PCS^d^) [49]	4
	Pain medication	Custom Analgesic Medication Use Survey	3 with branching logic to additional 7
	Health care utilization	Custom health care utilization survey for cLBP^e^	5 at baseline and 6 at all other timepoints
	Other measures		
	Assessment of affect	Positive and Negative Affect Schedule (PANAS) [55]	20
	Susceptibility to virtual reality treatment	Involvement subscale from the Immersive Tendency Questionnaire (ITQ – Involvement subscale) [54]	7
	Acceptability	System Usability Scale (SUS) [53]	10
	Perceived treatment assignment	Perceived Treatment Assignment survey	1

^a^DVPRS: Defense and Veterans Pain Rating Scale.

^b^PROMIS: Physical Function and Sleep Disturbance.

^c^VR: virtual reality.

^d^The 4 questions were selected from the PCS to decrease participant burden.

^e^cLBP: chronic lower back pain.

**Table 2:** Timeline of measures.

Measure	Pre-treatment (days –14 to 0)		Active treatment (days 1-56)		Postintervention (months 1-6 after the end of the study)			
	Baseline assessment (Day –14)	Pain surveys (Days –10, –7, –4, and 0)	Biweekly surveys	End of treatment (day 56)	Follow up 1 month	Follow up 2 months	Follow up 3 months	Follow up 6 months
DVPRS-I^a^ and DVPRS-II	X	X	X	X	X	X	X	X
PGIC^b^				X	X	X	X	X
PROMIS^c^ physical function	X			X	X	X	X	X
PROMIS sleep disturbance	X			X	X	X	X	X
Pain self-efficacy measures	X			X	X	X	X	X
PCS^d^	X			X	X	X	X	X
CPAQ-8^e^	X			X	X	X	X	X
Opioid use	X			X	X	X	X	X
Health care utilization	X			X				
Patient satisfaction					X	X	X	X
Device utilization			X	X				
VR^f^ usage data			X	X				
PANAS^g^	X			X				
SUS^h^				X				
ITQ^i^—involvement subscale	X							
Perceived treatment assignment								X

^a^DVPRS: Defense and Veterans Pain Rating Scale.

^b^PGIC: Patient’s Global Impression of Change.

^c^PROMIS: Physical Function and Sleep Disturbance.

^d^PCS: Pain Catastrophizing Scale.

^e^CPAQ-8: Chronic Pain Acceptance Questionnaire.

^f^VR: virtual reality.

^g^PANAS: Positive and Negative Affect Schedule.

^h^SUS: System Usability Scale.

^i^ITQ: Immersive Tendencies Questionnaire.

### Data Collection, Quality Control, and Confidentiality

All questionnaires will be completed by participants electronically via the REDCap Cloud platform. We will collect information at every stage of recruitment, randomization, and treatment in accordance with the CONSORT (Consolidated Standards of Reporting Trials) guidelines [57]. The Western Institutional Review Board approved this study. Given the safety of the device seen in past studies [7,31], Western Institutional Review Board did not deem that this study would require Data Safety and Monitoring Board oversight.

### Compensation

Participants will receive a total of US $150 (US $6 per completed survey) for their participation in the entire study. Two payments will be processed. The first payment will be distributed at the end of the 8-week program (US $126 possible; prorated) and upon return of their VR headset (prepaid shipping will be provided). The second payment will be distributed after the last follow-up survey (US $24 possible; prorated). All payments will be in the form of an Amazon eGift Card.

In addition to their monetary compensation, all participants will be eligible to receive a gift VR headset 6 months after their completion of treatment if they complete 16 or more of the 21 surveys administered during the active treatment phase, confirm their interest in receiving a VR headset, and return their VR treatment study headset.

### Safety Monitoring

Participants were provided with contact information and encouraged to contact as needed. Safety will be monitored by following up with participants for any adverse events they communicate to the support staff. Additionally, adverse experiences with using VR will be assessed using the question, “Did you experience any motion sickness or nausea while using VR?” on 4-point with 0 (Never), 1 (Sometimes), 2 (Often), and 3 (Always). Similar to prior work, VR side effects will be assessed at the end of treatment [32].

### Sample Size Determination

In terms of sample size considerations, a power analysis was performed using data from a recent at-home cLBP study that we conducted. DVPRS pain intensity scores were collected from 39 individuals at baseline, during, and immediately following a 21-day, skills-based VR intervention, and from 35 individuals at baseline, during, and immediately following an audio-only version of the 21-day program. The average difference score was 1.48 for the VR group and was 0.756 for the audio-only group (on an 11-point scale). Assuming an α level of .05 and 90% power, we would need 45 participants per group to observe a treatment × time interaction. In case of high attrition (40%), we will randomize at least 75 participants per group and if possible up to 90 participants per group.

### Statistical Analyses

#### General Approach

Checks of assumptions underlying statistical procedures will be performed and all corrective procedures will be applied as necessary. All analyses will involve 2-sided hypothesis tests, with α=.05 and adjusted for any multiple comparisons within the family of tests as appropriate.

Group equivalence will be assessed through univariate tests of association between treatment groups (EaseVRx/Sham VR) for all baseline demographic and clinical variables with chi-square and Kruskal–Wallis tests applied as appropriate. If statistically significant differences between groups are found for any variables (*P*<.05), those will be controlled for in the mixed models.

The data will be analyzed in a mixed-model framework (PROC GLIMMIX in SAS) with 3 explanatory factors: treatment group, time, and time × treatment group. Treatment group, EaseVRx versus Sham VR, will be specified as a between-subjects factor. Time will be specified as a within-subjects factor. The effect of interest will be the time × treatment group effect which tests whether the treatment group influenced the trajectory of the key variables over time.

The analytic method used will not involve imputing missing data for estimating the significance of the effects specified in the model. However, the predicted values from the estimated model will be used for reporting the findings. Given the safety of this treatment, there is no plan to conduct interim analyses.

#### Primary Analyses

The primary endpoint will be the time course of DVPRS-I Pain scale rating at baseline (defined as the average of 3 DVPRS-I Pain Scale ratings obtained during the 2 weeks before enrollment/randomization), at 8 weekly time points (twice per week) across the 8-week intervention, and immediately following the intervention. We will use a linear mixed model as described above.

#### Secondary Analyses

Several analyses will be proposed.

First, we will compare the PGIC scale at end of treatment and follow-ups.

Second, we will repeat similar analyses as above for 2 time points, baseline and immediately following the 8-week intervention for opioid drug use, PROMIS physical function, PROMIS sleep disturbance, PSEQ-2, PCS, and CPAQ-8.

Finally, we will repeat similar analyses as above for 2 time points, the day immediately following the 8-week intervention and 1 month after the intervention for DVPRS Pain Rating, opioid drug use, PROMIS physical function, PROMIS sleep disturbance, PSEQ-2, PCS, and CPAQ-8.

#### Exploratory Analyses

A number of exploratory analyses will be conducted, all of which envisage the above linear mixed modeling strategy with time points and variables as specified below.

First, we will assess Intervention × Time effects for a number of health-related outcome metrics (eg, number of steroid injections, emergency department visits, hospital admissions) at 2 time points, Day 9 and immediately following the 8-week intervention.

Second, we will repeat the above analyses for the period comprising the end of the 8-week intervention and at 3 and 6 months after the intervention.

Third, we will assess Intervention × Time effects for DVPRS Pain Rating, opioid drug use, PROMIS physical function, PROMIS sleep disturbance, PSEQ-2, PCS, CPAQ-8, Patient satisfaction, and PANAS for the periods comprising the 8-week intervention and at 1, 2, 3, and 6 months after the intervention. We will use a 2-factor ANOVA with intervention (EaseVRx vs Sham VR) as an independent groups factor and time as a dependent groups factor. Two-sided post hoc *t*-tests (adjusted for multiple comparisons) will be utilized to isolate the locus of any effects.

Fourth, we will examine the time course of changes in pain skills (eg, controlled breathing, meditation) from baseline, at the end of the 8-week intervention, and at 1, 2, 3, and 6 months after the intervention only in the EaseVRx group. We will use a one-factor repeated-measures ANOVA. Two-sided post hoc *t*-tests (adjusted for multiple comparisons) will be utilized to isolate the locus of any effects. When appropriate, we will also utilize more robust statistical approaches that better address missing data and do not assume distributional normality, such as bootstrapping.

In subsequent manuscripts, we will explore potential covariants of treatment response and possible mechanisms of actions.

## Results

The study was approved by the Western Institutional Review Board on July 2, 2020. The protocol (NCT04415177) was registered on May 27, 2020. Recruitment for this study was completed in July 2020 and data collection will remain active until March 2021. In total, 186 participants were recruited. Multiple manuscripts will be generated from this study. The primary manuscript will be submitted for publication in the winter of 2020.

## Discussion

### Protocol Overview

VR for chronic pain is an emerging area of behavioral medicine and science with heightened relevancy during the COVID-19 pandemic. Many people are environmentally isolated and in need of effective home-based pain care. This study protocol builds upon research that previously demonstrated that a 21-day behavioral medicine skills VR program effectively reduced chronic pain intensity and pain-related interference in activity, mood, sleep, and stress at the end of treatment. This study protocol addresses several unknowns that remain in the scientific literature for VR for chronic pain. First, the study will test a VR program of longer duration (56 days) and better aligns with the duration of current “gold-standard” behavioral medicine for chronic pain, typically over 8 weeks of treatment time. Second, the study will test treatment effects captured at the end of treatment and the *durability* of treatment effects measured at several distal posttreatment time points (months 1, 2, 3, 6). Third, the study will include a Sham VR, which will provide a visual treatment (2D nature scenes) that will control for the novelty of a headset device and visual stimuli while omitting active behavioral medicine skills training. The inclusion of the Sham VR group will also allow for exploration of the mechanisms of therapeutic VR. Fourth, a broad range of relevant metrics have been included to characterize the psychological response to VR and aid in the conduct of responder analyses and identification of subgroups; results could inform the development of future tailored immersive therapeutics or study designs. Fifth, all study headsets will capture participant use data, thereby allowing for the quantification of participant engagement and calculation of treatment dose thresholds associated with treatment effects. Sixth, the study will capture analgesic medication use and data on health care utilization specific to back pain; these data will allow for the conduct of exploratory analyses examining the impact of VR on these factors for the subset of participants using these treatments. Seventh, the study will occur within the context of the COVID-19 pandemic and will inform self-administration of home-based VR and engagement during COVID-19 specifically.

The study design’s strength is that it will be conducted remotely and untethered from the medical system. This design will increase the ecological validity of data derived from a home-based, national, pragmatic sample of people with cLBP who will self-treat in their home environment. Additional aspects of methodological rigor include participant blinding and randomization to the treatment group.

### Limitations

The key limitations of this study protocol include the following. First, all data will be either self-reported by the study participant or collected by the device (eg, use data for frequency and duration). Because the study is pragmatic and will include a national sample, we will not verify medical diagnoses or prescribed pain medication types and doses. Second, the study is specific to cLBP and findings may not generalize to other pain conditions. However, we note that people with cLBP often report having 2 or more comorbid pain conditions (Darnall et al, unpublished). As such, chronic back pain is not often experienced in isolation.

Digital behavioral health treatment studies typically report relatively low treatment engagement rates among participants with rates ranging between 20% and 60% [32,58-60]. While prior research evidenced good engagement for therapeutic VR for chronic pain, engagement rates for a 2D Sham VR are unknown and we may risk disparate engagement rates between the 2 treatment groups. While the study team has endeavored to minimize such discrepancy by enhancing the Sham VR’s face validity, we anticipate some treatment group discrepancy would naturally occur if one treatment is experienced broadly as less rewarding or effective.

Our plan to enroll a national sample over the internet lends a mix of strengths and limitations. Participants recruited via the internet are likely to be more technologically savvy than the general population seeking medical care from a health care system. It could be argued that our study results may not generalize to people who are less likely to engage with the internet and technology. However, we also note that treatment studies that are conducted within traditional medical settings typically involve more in-person contacts and enhanced placebo effects (ie, halo effect) that would be likely to yield more positive treatment expectations and outcomes. We underscore that our study design will not benefit from medical setting placebo effects.

Finally, aligning with prior work, data on adverse effects will be collected at the end of the study. We acknowledge that these methods introduce the potential for recall bias. However, previous study participants reported easily recalling adverse experiences at the end of the study due to their specificity and salience (eg, cybersickness) [32].

### Conclusions

This study will be one of the most rigorous in assessing the impact of self-administered VR therapy in community-based individuals with chronic lower back pain and the first to use a placebo VR therapy program. Its remote design will allow it to be completed during a global pandemic in a pragmatic and nationally representative sample. This will also be the first study to assess VR therapy’s durability for chronic pain over a 6-month posttreatment follow-up period. Results from this study will provide critical data on how individuals with chronic lower back pain may use self-administered VR therapy at home for symptom management and functional improvement.

## Abbreviations

CDC: Centers for Disease Control and Prevention
cLBP: chronic lower back pain
CMS: Centers for Medicare & Medicaid Services
CPAQ: Chronic Pain Acceptance Questionnaire
DVPRS: Defense and Veterans Pain Rating Scale
ITQ: Immersive Tendencies Questionnaire
MME: morphine milligram equivalent
PANAS: Positive and Negative Affect Schedule
PCS: Pain Catastrophizing Scale
PGIC: Patient’s Global Impression of Change
PHQ: Patient Health Questionnaire
PROMIS: Physical Function and Sleep Disturbance
PSEQ: Pain Self-Efficacy Questionnaire
SUS: System Usability Scale
