# Correction: Self-Administered Behavioral Skills-Based At-Home Virtual Reality Therapy for Chronic Low Back Pain: Protocol for a Randomized Controlled Trial

**DOI:** 10.2196/27652

**Published:** 2021-02-12

**Authors:** Laura M Garcia, Beth D Darnall, Parthasarathy Krishnamurthy, Ian G Mackey, Josh Sackman, Robert G Louis, Todd Maddox, Brandon J Birckhead

**Affiliations:** 1 Research and Development AppliedVR Inc Los Angeles, CA United States; 2 USC Creative Media and Behavioral Health Center Los Angeles, CA United States; 3 Department of Anesthesiology Perioperative and Pain Medicine Stanford University School of Medicine Stanford, CA United States; 4 C T Bauer College of Business Houston, TX United States; 5 Division of Neurosurgery Pickup Family Neurosciences Institute Hoag Memorial Hospital Newport Beach, CA United States; 6 Division of Health Services Research Department of Medicine Cedars-Sinai Health System Los Angeles, CA United States

In “Self-Administered Behavioral Skills–Based At-Home Virtual Reality Therapy for Chronic Low Back Pain: Protocol for a Randomized Controlled Trial” (JMIR Res Protoc 2021;10(1):e25291), the authors noted five author names missing middle initials.

In the originally published paper, the list of authors appeared as follows:

Laura Garcia, Beth Darnall, Parthasarathy Krishnamurthy, Ian Mackey, Josh Sackman, Robert Louis, Todd Maddox, Brandon Birckhead

The list has been corrected as follows:

Laura M Garcia, Beth D Darnall, Parthasarathy Krishnamurthy, Ian G Mackey, Josh Sackman, Robert G Louis, Todd Maddox, Brandon J Birckhead

The correction will appear in the online version of the paper on the JMIR Publications website on February 12, 2021, together with the publication of this correction notice. Because this was made after submission to PubMed, PubMed Central, and other full-text repositories, the corrected article has also been resubmitted to those repositories.

